# Screening Blind Spot: Missing Preterm Infants in the Detection of Congenital Hypothyroidism

**DOI:** 10.3390/ijns11020037

**Published:** 2025-05-13

**Authors:** Ashleigh Brown, Paul Hofman, Dianne Webster, Natasha Heather

**Affiliations:** 1 Starship Child Health, Te Whatu Ora Te Toka Tumai Auckland, Auckland 1023, New Zealandnheather@adhb.govt.nz (N.H.); 2 Liggins Institute, University of Auckland, Auckland 1023, New Zealand; 3 LabPlus, Te Whatu Ora Te Toka Tumai Auckland, Auckland 1148, New Zealand

**Keywords:** congenital hypothyroidism, preterm, screening, newborn screening, low birth weight

## Abstract

Congenital hypothyroidism (CH) is a critical condition in infancy where early detection is vital for optimal development. This study aimed to evaluate the sensitivity of Aotearoa New Zealand’s Newborn Metabolic Screening “Low Birth Weight” protocol for detecting CH in preterm infants. A 10-year audit was conducted on 2935 preterm infants (<2000 g or ≤34 weeks gestation) screened within NICUs or SCBUs in the Auckland region. The study assessed both screen-detected and clinically detected cases of CH. Data were collected from screening and clinical records to evaluate the sensitivity and reliability of the current protocol. The audit identified 19 cases of primary CH, with a 1:154 incidence. Thirteen cases met the criteria for inclusion in the audit. Just over half of the eligible cases (7/13) were screen-detected, while the remaining were detected clinically, suggesting limitations in screening sensitivity. The analysis revealed that the protocol missed permanent as well as transient cases, and that biochemical severity was not predictive of permanence. A revised screening protocol was developed and commenced in July 2024.

## 1. Introduction

Primary congenital hypothyroidism (CH) is the most common disorder detected on newborn metabolic (bloodspot) screening and a common preventable cause of intellectual disability worldwide [1,2,3]. Primary CH causes thyroid hormone deficiency due to an absent, ectopic, or under-functioning thyroid gland. While the overall incidence of CH is approximately 1:2000, it is much higher among preterm infants, with reported rates ranging from 1:100 to 1:150 in very preterm infants [2,4,5,6,7,8]. Whilst CH in preterm babies is frequently transient, a significant proportion of preterm infants have permanent CH [7] and thyroid-stimulating hormone (TSH) concentrations at diagnosis do not appear to be a reliable predictor of permanence [9]. The aetiology of the increased frequency of CH in preterm infants remains unclear but is hypothesised to be due to several factors, including immaturity of the hypothalamic–pituitary–thyroid axis, medications (steroids, dopamine), non-thyroidal illness (e.g., respiratory distress syndrome), and iodine deficiency [5,8,10,11]. The maturation of thyroid function in preterm infants differs from that of term infants, necessitating a revised screening protocol in this population, which is discussed in detail elsewhere [7,8].

In Aotearoa New Zealand, nearly 8% of infants are born preterm (before 37 weeks of gestation) [12]. Prematurity is the most common reason for admission to a level III neonatal unit, with very preterm babies (<32 weeks gestation) accounting for 25–30% of admissions. Māori and Pasifika infants are over-represented in the level III neonatal unit, making up 21.3% and 11.8% of admitted infants, respectively, compared to their proportion of 16.5% and 8.5% in the general population [13,14].

Over the past decade, screening programmes have become increasingly aware that preterm infants with CH are likely to have normal initial screening results due to a delayed rise in TSH concentrations [15,16]. To improve the reliability of CH screening, the New Zealand screening programme introduced a protocol (‘the Low Birth Weight (LBW) protocol’) for repeat sample collection from low birth weight (<1500 g) infants in 2007. All participating infants had their first screening sample at 48–72 h of age, infants with a low birth weight of <1500 g had a second screening sample at 14 days of age, and extremely low birth weight infants (<1000 g) had a third screening sample at 28 days of age. Despite over 10 years of experience, the performance of the LBW protocol has never been comprehensively evaluated.

Our objective was to evaluate the screening sensitivity of this protocol by auditing both the screening and clinically detected primary CH diagnoses in preterm infants in the Auckland region over a 10-year period. Other aspects such as screening specificity, aetiology, and disease severity were out of the scope of this study.

## 2. Materials and Methods

### 2.1. Aotearoa New Zealand Newborn Metabolic Screening Process for Low Birth Weight Infants (<1500 g) During Audit Timeframe

**Figure 1:**
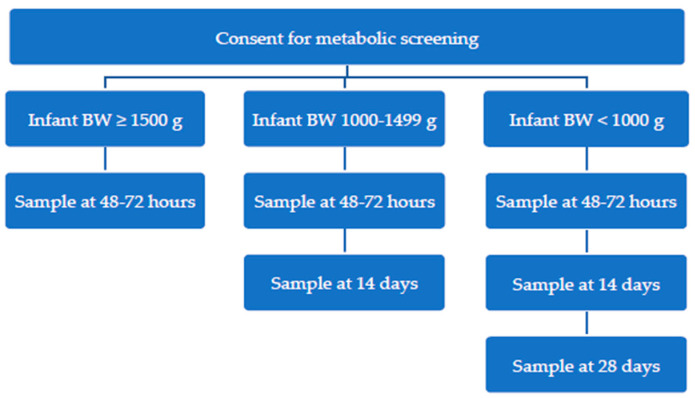
New Zealand newborn screening algorithm for congenital hypothyroidism during audit timeframe; BW = birth weight.

Approximately 60,000 infants undergo Newborn Metabolic Screening in New Zealand each year, with very high (>99%) population coverage. Infants born with a birth weight of less than 1500 g followed the “Low Birth Weight Protocol”, with approximately 500 infants screened nationwide under this protocol annually. The protocol included an initial screening at 48–72 h, a second at 14 days for infants <1500 g, and a third sample at 28 days for those born <1000 g (see Figure 1) [17]. Blood samples were collected onto specialised collection paper and sent to the single national screening laboratory at LabPlus, Auckland, New Zealand. Screening samples were analysed for markers of a full metabolic screening panel [17], including whole blood TSH concentration via AutoDELPHIA (Perkin-Elmer, now Revvity). Results were reported on the next working day after the sample was received.

Elevated TSH results prompt notification to the responsible neonatologist for serum thyroid function tests. TSH thresholds for screening vary by postnatal age due to the physiological TSH surge immediately after birth, which rapidly declines over the first few days of life (Table 1).

Blood spot TSH thresholds are given in whole blood units. A conversion factor of 2.2 is typically used to estimate serum units, based on an assumed haematocrit of 0.55. However, preterm and low birth weight infants often have much lower haematocrits, which can result in falsely elevated TSH results when using this standard conversion [18]. Thresholds have since been adjusted further due to changes in the assay used and refinement of thresholds by postnatal age.

### 2.2. Audit Methodology

The Auckland region has a population of 1.5 million people, with an annual birth rate of approximately 22,000 live births per year [14,19]. Very preterm infants are cared for at two neonatal intensive care units and one special care baby unit across the region, providing level III and level II care, respectively. Starship Child Health provides a single regional referral service for infants and children with endocrine conditions, including CH.

Infants participating in newborn screening born at or before 34 weeks of gestation (<34 + 1) with a birth weight of <2000 g in the Auckland region between 1 January 2009 and 31 December 2018 were included in the audit. Non-Auckland residents, those born >34 weeks gestation, and any infants who died during their admission were excluded from the audit. Gestational age and birth weight parameters were wider than standard preterm screening criteria (<32 weeks gestational age and/or <1500 g birth weight) to assess if standard parameters were adequately detecting affected infants.

Infants with screen-detected CH were identified from newborn screening records. The Starship Child Health Paediatric Endocrinology Department’s Thyroid Clinic patient database was cross-referenced against the newborn screening database to identify additional infants being followed up for CH that were not identified by screening.

Data extraction was performed by the laboratories at each hospital site to identify any infant born at <34 + 1/40 gestation within the audit timeframe at all sites that ever had serum thyroid function tests performed during their admission. Any infant with a serum TSH of ≥10 mU/L at any time was cross-referenced with the newborn screening data, Clinical Portal, and the Starship Paediatric Endocrinology Department’s patient database to identify any remaining infants with congenital hypothyroidism that were not detected by screening and lost to follow-up by the local service. The threshold of 10 mU/L for serum TSH was selected as a consensus, as infants with a TSH persistently above this level should be investigated further, if not initiated on levothyroxine supplementation [20].

Clinical data for each identified infant were examined manually to evaluate the type and magnitude of thyroid dysfunction, method of diagnosis, investigations, co-morbidities, exposure to intravenous contrast or topical antiseptics containing iodine, type and duration of treatment, and outcomes.

For the purpose of the audit, a case was defined as an infant with biochemical results supporting a diagnosis of primary congenital hypothyroidism (persistently elevated TSH concentration) and was initiated on thyroxine treatment. Severe cases were defined as serum TSH level ≥ 50 mIU/L in diagnostic testing, and moderate CH was defined as a serum TSH level ≥ 20 mIU/L. Infants treated with thyroxine due to sick euthyroid or central hypothyroidism were excluded from the audit, as these conditions are not targeted through our newborn screening approach. We defined “clinically detected” as cases detected through serum thyroid function tests and unscheduled screening cards that would not have otherwise been detected by the screening programme protocol.

### 2.3. Statistical Analysis

All patient data were collated in a CSV sheet in Microsoft Excel. Data were sorted by confirmed diagnosis of CH and the detection method. A simple count was performed to identify infants detected by screening compared to infants detected clinically. Each individual data set was then examined manually to describe characteristics of cases.

## 3. Results

An audit indicates that 2935 babies weighing less than 2000 g or born at 34 weeks gestation or earlier were screened in NICUs or SCBUs in the Auckland region. Nineteen cases of primary CH were identified, leading to a CH incidence of 1 in 154.

Complete data were available in 13/19 cases, which formed the basis of our audit. These cases are summarised in Table 2. Just over half the CH cases (7/13) were screen-detected, and the remainder (6/13) were clinically detected, indicating limited sensitivity of the current screening approach. An additional six cases of primary CH (four screen-detected and two clinically detected) were excluded from the audit due to living outside of the Auckland region and having incomplete data available for review. No additional cases were identified by cross-referencing regional laboratory results or from the paediatric endocrinology patient database.

All screen-detected cases were identified from samples collected at 2 or 4 weeks, following normal bloodspot TSH concentrations at 48–72 h. The majority of CH cases had biochemically moderate to severe thyroid disease (TSH ≥ 20 mIU/L to ≥50 mIU/L, respectively) at presentation. Most cases (10/13) had transient disease and were able to cease thyroxine replacement between 2 and 3 years of age. Biochemical severity at diagnosis was not predictive of permanent disease. None of the cases had been exposed to intravenous contrast.

Clinically detected cases were typically older and had milder biochemical disease at detection than screen-detected cases. The reasons for clinical detection were varied and included additional unscheduled screening cards, primary CH incidentally detected through pituitary function testing, investigation of dysmorphisms, and feeding problems. As with screen-detected cases, biochemical severity was not predictive of permanent disease.

## 4. Discussion

The goal of newborn metabolic screening is to facilitate better outcomes through the early identification of congenital disorders. It is not possible to detect all cases through screening, and there are numerous factors (e.g., prematurity, intravenous nutrition, transfusions) that can increase the likelihood of false-negative and false-positive screen results in NICU babies. Newborn screening has been adapted to better suit the NICU population through the introduction of additional samples and modified analyte thresholds. The global consensus supports repeat testing for low-birth-weight infants; however, there is no agreed-upon schedule for the timing of the samples, and it varies around the world. The main arguments for and against repeated testing balance early detection and treatment versus the cost of extra testing and potentially unnecessary subsequent clinical intervention.

Repeat screening sample collections in NICU babies can increase disorder detection and reduce referrals for diagnostic testing. For preterm and low-birth-weight infants in New Zealand, a complete “newborn screen” is considered a series of related tests, rather than a combination of two or three individual screens. Collection algorithms are designed to be simple enough to follow, as the screening programme is unable to send reminders, and it is assumed that all expected bloodspot samples will be received. The previous protocol was based on low birth weight (<1500 g) as a proxy for prematurity, as when discussed in 2007, this was felt to be a more objective, readily available measure. However, combined birth weight and gestational age criteria are commonly utilised in existing NICU protocols, suggesting that gestational age could similarly be incorporated into the metabolic (bloodspot) screening protocol. Victoria, Australia, recently amended their preterm and sick infant protocol and added gestational age <32 weeks and/or birth weight <1500 g. The addition of gestational age captured an extra 5% of infants and is felt not to have a significant influence on the number of repeat samples required [21].

As expected, we observed a greater than 10-fold higher incidence of primary CH in very preterm babies compared with the overall population, and very poor reliability of the initial sample in detecting CH. It was reassuring to note that the most severe cases (TSH ≥ 50 mIU/L) were screen-detected through the current protocol, as a result of samples collected at either 2 or 4 weeks postnatally. A cluster of cases in very preterm babies who were just under or above the 1500 g birth weight threshold suggested that reliability may be increased by incorporating gestational age as an ‘and/or’ criteria with birth weight (i.e., a protocol for re-collection in babies <32/40 and/or <1500 g), without the need to increase the weight cut-off. Of concern, several clinically detected permanent and/or biochemically moderate CH cases (TSH ≥ 20 mIU/L) were associated with a TSH rise which occurred later than the final screening sample, but prior to NICU discharge. Although the increased number of screen-detected cases cannot reliably be predicted, this suggests that screen detection of persistent or permanent CH would be improved by the addition of a final pre-discharge screening sample.

It is accepted that many preterm infants will have TSH blood concentrations within range on their initial screening test at 48–72 h of age, regardless of underlying congenital thyroid defects [15,22]. Due to the delayed TSH elevation in preterm infants, many programmes worldwide do additional screening in this group. There is no consensus on the optimal timing of repeat screening sample collection. Our New Zealand Low Birth Weight re-collection guideline was largely consistent with Australian programmes [2], but involves fewer re-collections than other countries (Table 3).

Vincent et al. [26] argued against re-screening in VLBW infants, as in their study of 465 infants screened initially at 2–5 days and again at 6 weeks of age, they only identified four cases of permanent primary congenital hypothyroidism, and all four were detected in the initial screening sample. Applying this strategy to the 13 cases in our audit, none of our cases were detected on the first screen, and it would have delayed diagnosis in seven infants who were detected on screens at 2 and 4 weeks of age. A study in Rhode Island [4] demonstrated that the delayed TSH elevation seen in preterm infants can be transient, with serum TSH normalising at a mean of 51 days of age. They hypothesised that re-screening may not be necessary; however, their neurodevelopmental follow-up found an increased incidence of infants with a head circumference <10th percentile at 18 months of age in the delayed TSH elevation group. Suboptimal head circumference measurements that persist beyond the initial neonatal period are associated with poorer neurodevelopmental outcomes [27]. In term infants, prompt normalisation of TSH and free T4 within the first two weeks of life is correlated with improved full-scale IQ [28]. Regardless of the permanence of disease as well as the difficulty in predicting transient disease, it would seem justified to treat infants with a clinical picture of primary hypothyroidism. There are few long-term follow-up studies of preterm infants with delayed TSH elevation, and many of these infants receive levothyroxine supplementation, making long-term outcomes difficult to assess. Preterm infants as a population are already vulnerable to long-term disability and neurocognitive deficits [29], so it would seem prudent that all care should be taken to minimise further insults.

Current expert guidance from the international Clinical Laboratory Standards Institute (CLSI) is that a further newborn screening sample should be collected from all infants < 34/40 gestation or <2000 g birth weight, either at 28 days of age or discharge [30]. The European Society of Paediatric Endocrinology (ESPE) recommends that repeat specimen collection criteria be expanded further, i.e., that a repeat routine specimen be collected at 2 weeks postnatal age in all ill, preterm (<37 weeks gestational age), low-birth-weight infants (<2500 g) or multiple births, particularly same-sex twins, admitted to an NICU [9]. Since this audit was undertaken, the American Academy of Pediatrics (AAP) have updated guidance (in 2023) to recommend a repeat specimen at 2–4 weeks of age for infants < 32 weeks gestational age or <1500 g, and a further follow-up specimen if infant has not reached 36 weeks postmenstrual age at the time of the second sample [31].

### New ‘Preterm Metabolic Bloodspot Screening Protocol

**Figure 2:**
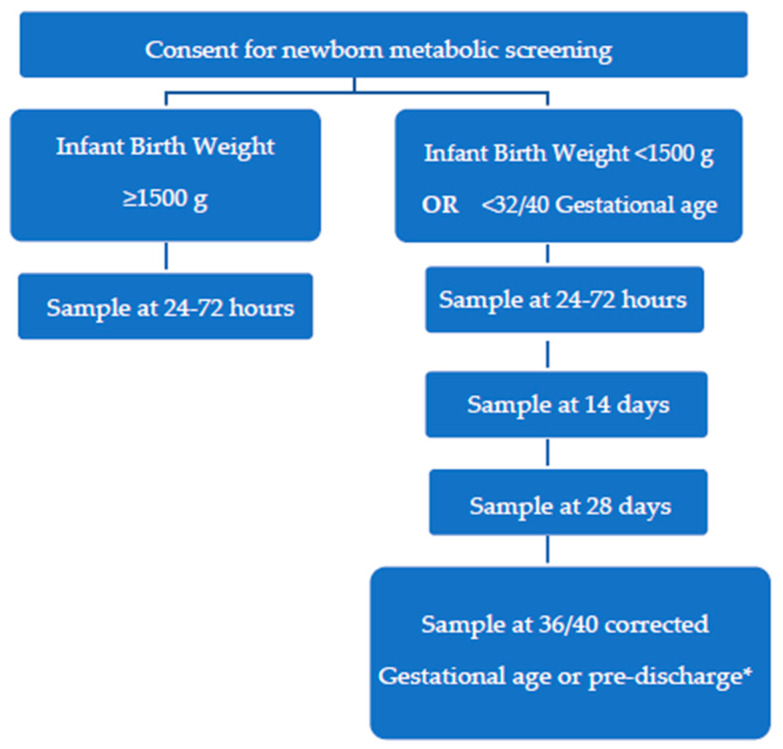
‘Preterm Metabolic Bloodspot Screening Protocol’; BW = birth weight. * Unless there has already been a bloodspot sample collected within 2 weeks.

The audit group undertook a stakeholder consultation process including neonatologists, regional paediatricians, paediatric endocrinologists, and screening laboratory scientists. The consensus was acceptance of the need to increase screening of preterm infants and that adding gestational age criteria in addition to weight-based criteria would not cause confusion or difficulty. The overwhelming preference was for a simple algorithm that was easy to follow. A new, simplified, and extended screening algorithm was developed (Figure 2), taking into account changes to sample timing and TSH thresholds that had occurred in the time since the audit occurred and analysis of results. This algorithm was presented to the Newborn Metabolic Screening Technical Working Group, which is responsible for technical oversight of the programme. The updated screening protocol was accepted in March 2024. The ‘Preterm Metabolic Bloodspot Screening Protocol’ replaced the previous ‘Low Birth Weight Protocol’ for infants with a birth weight under 1500 g from 1 July 2024.

The amendment of the protocol brings our practice in line with the recent AAP guideline recommending retesting of infants <1500 g and/or <32 weeks gestation [31] and incorporates the CLSI recommendation for follow-up at 28 days of age or discharge [30]. We did not find evidence to support the CLSI recommendation to broaden the inclusion criteria to <34/40 gestational age and birth weight of <2000 g. It is also not as broad as the European Society of Paediatric Endocrinology (ESPE) recommendation for further expansion of repeat specimen collection criteria, i.e., that a repeat routine specimen be collected at 2 weeks postnatal age in all ill, preterm (<37 weeks gestational age), low-birth-weight infants (<2500 g) or multiple births, particularly same-sex twins, admitted to an NICU [9]. Our protocol does not differentiate between well and unwell infants, and we no longer have different testing requirements for extremely low-birth-weight infants (<1000 g) in order to simplify the criteria for clinicians. Under the new preterm protocol, we estimate that an additional 1029 samples per year would be collected, including two or three repeat samples from 144 babies who are <32/40 but not <1500 g. This represents an increase of less than 2% of all national samples and a minimal additional cost. We intend to review this protocol in two years.

Neither proposed change (i.e., the addition of gestational age criteria and an additional pre-discharge sample) is anticipated to have a negative impact on screening for disorders other than CH. Prematurity and illness both commonly lead to low T-Cell receptor excision circles (TREC) and high 17-hydroxy-progresterone (17-OHP) screening levels, and borderline-positive screen results for severe combined immunodeficiency (SCID) and congenital adrenal hyperplasia (CAH), respectively [32,33]. If the results do not normalise within the routine testing schedule, the screening programme may request a further bloodspot sample or recommend diagnostic testing, but the number of diagnostic referrals is kept as small as feasible. For CAH, the number of false-positive screens in sick and preterm babies has been dramatically reduced by reflexing samples with high 17-OHP levels to a highly specific second-tier test (steroid profile) [34]. For SCID, the protocol considers that a baby with a normal TREC result on any previous sample to have had a normal screen for SCID, even though subsequent TREC levels may drop below the threshold if the baby becomes more unwell. As such, the collection of additional samples may, in fact, (minimally) reduce the number of diagnostic referrals for SCID and CAH.

Limitations of this audit are that it was restricted to infants within the Auckland region; however, it is Aotearoa New Zealand’s largest city, comprising one-third of New Zealand’s population, and includes two of the six neonatal intensive care units in the country. Our study was limited to infants under 34 weeks and 2000 g, so we were unable to comment on screening performance in ill, late preterm infants, or same-sex twins.

## 5. Conclusions

Findings that the low-birth-weight screening protocol was missing just under half of preterm and very low-birth-weight infants treated for primary CH prompted a stakeholder consultation and subsequent change in process. We have incorporated gestational age criteria and the addition of a pre-discharge sample to improve the sensitivity of the screening. This change is not anticipated to significantly increase the cost of screening or the workload of laboratory staff, but it provides a safeguard to minimise missed cases. Given their increased risk for long-term disabilities and neurocognitive challenges, all care should be taken to prevent further harm to preterm infants. Our findings highlight a gap in preterm screening in Aotearoa New Zealand and possibly globally. Further modifications to preterm screening could include a lowering of the TSH thresholds to detect more cases; however, it appears that the timing of the sampling relative to the postnatal age of the infant may be more important. The delayed rise in TSH concentrations in preterm infants suggests the need for further expansion of preterm screening, and this area would benefit from a standardised approach. Further investigation into the aetiology of this marked increase in CH in this population is warranted.

## Figures and Tables

**Table 1:** Newborn screening TSH thresholds during the audit timeframe.

Protocol prior to 1 January 2013:	
	TSH < 15 mIU/L = Normal TSH ≥ 15 mIU/L = Follow-up specimen required
Protocol from 1 January 2013:	
Age ≤ 14 days:	TSH < 15 mIU/L = Normal TSH ≥ 15 mIU/L = Follow-up specimen required
Age > 14 days:	TSH < 8 mIU/L = Normal TSH ≥ 8 mIU/L = Follow-up specimen required

**Table 2:** Summary of audit findings.

Sex	Gestational Age at Birth	Birth Weight (g)	Ethnicity	Screen Positive Sample	Reason for Thyroid Function Tests	Age at Diagnosis	Screening Whole Blood TSH (mIU/L)	At Diagnosis		Outcome (Permanent, Transient)	Age Ceased Thyroxine	Co-Morbidities
	(Weeks + Days)							Serum TSH (mU/L) Ref: 0.4–16 mU/L	Serum Free T4 (pmol/L) Ref: 10–40 pmol/L			
**Clinically detected**												
Male	28 + 3	830	Samoan	Fourth	Third screen borderline for amino acid breakdown disorder, fourth sample requested	6 weeks	8	18.2	17	Transient	2 years	Very preterm, ambiguous genitalia with imperforate anus, ELBW, Apnoea of prematurity, RDS, jaundice
Male	28 + 3	1350	Samoan	Third	Unscheduled third screening sample	5 weeks	18	33	6.2	Transient	2 years	Very premature, RDS, Apnoea of Prematurity, RoP
Female	29	980	New Zealand European		Maternal Hypothyroidism	6 weeks		39	12.5	Transient	2 years	Very preterm, RoP, AOP, RDS, jaundice,
Male	30	1600	Chinese		Feeding problems	12 weeks		11.9	14	Transient	18 months	Very preterm
Male	31	1490	Indian		Pituitary screening	8 weeks		19.7	17	Permanent		Very preterm, 45XO/46XY, Hypospadias
Female	32	1420	New Zealand European		Dysmorphism, macrocephaly, and seizures	6 weeks		10	15	Permanent		Moderate preterm, RDS, later diagnosed with GDD
**Screening detected**												
Female	24 + 3	650	New Zealand European	Third		5 weeks	14	41	9.6	Transient	2 years	Extreme preterm, ELBW, CLD, Apnoea of prematurity, RoP, PDA
Male	27 + 5	1105	Fijian	Second	Borderline second screen, third sample requested	4 weeks	74	>100	3.7	Transient	2.5 years	Extreme preterm, VLBW, RDS, CLD, left inguinal hernia, CMV
Male	28 + 6	900	Cook Island Māori	Second		3 weeks	13	39.9	12.7	Transient	2.5 years	Very preterm, ELBW, RDS, hypospadias with chordee
Female	29 + 5	720	Cook Island Māori	Second		3 weeks	78	138	6	Permanent		Very preterm, ELBW, RDS, CLD, Twin 2
Female	30 + 1	1455	Chinese	Second		4 weeks	22	49	9.9	Transient	5 years	Very preterm, VLBW, RDS, jaundice, maternal GDM
Female	30 + 5	1065	New Zealand Māori	Second		17 days	32	>100	3.2	Transient	3 years	Very preterm, VLBW, apnoea of prematurity, AOP, PDA
Female	32 + 1	1235	Samoan	Second		4 weeks	19	42.2	10.9	Transient	6 months	Moderate preterm, VLBW, RDS, SGA

AOP = anaemia of prematurity; CMV = cytomegalovirus; CLD = chronic lung disease; ELBW = extremely low birth weight; GDD = global developmental delay; GDM = gestational diabetes mellitus; PDA = patent ductus arteriosus; RDS = respiratory distress syndrome; RoP = retinopathy of prematurity; SGA = small for gestational age; VLBW = very low birth weight.

**Table 3:** Comparison of international newborn screening protocols.

State or Country	First Sample	Second Sample	Third Sample
Aotearoa New Zealand [17]	48–72 h	14 days if BW < 1500 g	28 days if BW < 1000 g
Queensland, Australia [2]	48–72 h	14 days if BW < 1500 g	28 days if BW < 1000 g
Western Australia, Australia [2]	48–72 h	14 days if BW < 1500 g	28 days if BW < 1000 g
South Australia, Australia [2]	At or near 48 h	10 days if BW < 1500 g	30 days or discharge if BW < 1500 g
Victoria, Australia [2,21]	36–72 h	4 weeks or discharge if BW < 1500 g or GA < 32 weeks	
New South Wales, Australia [2]	48–72 h	1 month if BW < 1500 g or GA < 30 weeks	
British Columbia, Canada [23]	24–48 h	21 days or day of discharge if BW < 1500 g	
United Kingdom [24]	5 days	28 days or day of discharge if GA < 32 weeks	
Wisconsin, USA [15]	24–48 h	14 days if BW < 2000 g or GA < 34 weeks	30 days for all infants
Japan [25]	5–7 days	4 weeks or body weight reaches 2500 g or at discharge if BW < 2000 g	

BW = birth weight, GA = gestational age at birth.

## Data Availability

Data supporting the findings of the study are not publicly available in order to protect participant privacy and confidentiality. Participant consent for data access is restricted to screening programme monitoring and quality improvement. Interested parties may contact the authors for specific requests, subject to review and approval.
